# Multiple Effects of Egg Weight, in Ovo Carbohydrates, and Sex of Birds on Posthatch Performance in Broilers

**DOI:** 10.3390/vetsci9090491

**Published:** 2022-09-10

**Authors:** Virág Ács, Szilvia Áprily, József Nagy, László Kacsala, János Tossenberger, Nóra Katalin Szeli, Veronika Halas

**Affiliations:** 1 MTA-MATE Mycotoxins in the Food Chain Research Group, 7400 Kaposvár, Hungary; 2 Department of Farm Animal Nutrition, Hungarian University of Agriculture and Life Sciences Kaposvár Campus, 7400 Kaposvár, Hungary; 3 Department of Precision Farming and Animal Biotechnology, Hungarian University of Agriculture and Life Sciences Kaposvár Campus, 7400 Kaposvár, Hungary; 4 AVI-VET Ltd., 7400 Kaposvár, Hungary

**Keywords:** broiler, nutrition, in ovo feeding, carbohydrates, glucose, embryo, body weight, carcass, meat yield

## Abstract

**Simple Summary:**

Future technologies in poultry nutrition must support the growth, gut health, and energy status of the birds. One of these technologies is in ovo nutrition, in which nutrients enter the egg by a needle puncture without any harm to the embryo. Amino acids, vitamins, and even carbohydrates can be injected into various parts of the egg, mainly during the last days of hatch. Carbohydrates, as a primary energy source, are crucial for the avian body, especially on the day of hatch, when the carbohydrate resources of the egg yolk do not meet the needs of the embryo. In our study, a carbohydrate mixture composed of disaccharides and glucose was tested to examine the performance and carcass characteristics of broiler chickens. It can be concluded that a carbohydrate mixture tends to reduce hatchability; however, the correct quantity of fluids, injection day, and osmolality of the mixture still need to be specified. In addition, many other factors can determine performance, such as the hatching day, sex, and the size or weight of the egg. On the other hand, this technique may improve carcass traits. Further studies are needed to refine the method to avoid embryo death.

**Abstract:**

Chickens raised for their meat (*Gallus gallus domesticus*) tend to have a critical phase of life right after hatching due to the management of modern production systems. Early nutrition strategies such as in ovo intervention can be an alternative means to support growth and gut health by compensating for the energy deficit after pipping out of the egg. In the current study, 1200 Ross 308 eggs were used to examine the effects of a complex carbohydrate solution of disaccharides and glucose applied in ovo on hatchability, the hatching time of different-sized eggs, and the development, performance, and carcass characteristics of broilers of both sexes. The eggs were divided into three treatment groups: intact (NT), in ovo saline (ioS), and in ovo carbohydrate mixture (ioCH). The incubation protocol was performed according to the recommendations of Aviagen (2019), and the in ovo process was carried out on day 17 by manually injecting 0.5 mL of the solutions into the amniotic fluid. After hatching, the birds were kept in floor pens until day 35 and fed ad libitum in a three-phase feeding program. Body weight, average daily weight gain, feed intake and conversion, and carcass characteristics were measured during the trial. In ovo carbohydrates reduced hatchability by 15%, while growth performance and the weight of thigh and breast muscle were enhanced significantly (*p* < 0.05) compared with ioS as a possible outcome of carbohydrate-to-muscle satellite cell proliferation and protein accumulation. However, further study is needed to refine the in ovo carbohydrate supplementation method to minimize the mortality of embryos during hatching.

## 1. Introduction

Selection to lower the slaughter age and raise the meat yield of broiler chickens has resulted in the rapid growth of the poultry industry over the past 60 years [1]. This trend may continue if novel findings in biotechnology and nutrition can exploit the genetic potential of the birds. Therefore, breeding companies have directed their research to examine the performance of broiler lines and improve carcass yield within a relatively short period of time. Investigating the developmental mechanisms of the broiler embryo is particularly important because the embryonic phase takes up to 21 days and represents 35–40% of the total lifespan [2].

During this process, the embryo is dependent on the albumen and the yolk sac [3] to provide energy and nutrients for the prenatal phase, which has a moderate ability to support the rapid growth of broiler chickens [4]. Complex nutrients, such as proteins, fats, and carbohydrates, are mobilized from the yolk sac and converted into amino acids, fatty acids, and glucose in the liver during embryogenesis and transported to tissues via circulation [5]. Glucose, a simple sugar, is the primary energy source [6], which, unlike fat-generated energy, requires a minor amount of oxygen [7]. Protein and fat are the main sources of energy because the egg is only 0.3–0.4% carbohydrates [8], which do not meet the needs of the late-term embryo. Homeostatic regulation requires different metabolic processes to generate energy such as gluconeogenesis, in which the glycol is only available from lipolysis and proteolysis [9]. At hatch, broiler embryos prefer glucose to fatty acids because it provides more energy than lipid catabolism when oxygen reserves are limited [10].

The low level of carbohydrates in the yolk [11] may also lead to embryonic ketosis, the forming of ketone bodies from acetyl coenzyme A [12]. For rapid muscle contractions to perforate the eggshell at the time of emergence and to prevent ketone body accumulation [11], the supply of glucose remains immensely important. In case of a deficiency, primary energy reserves are tapped for maintenance, causing reductions in the pectoralis muscle and lower organ weight [13] along with decreased body weight (BW) and performance [14].

Adding glucose to the drinking water to suppress gluconeogenic activity, support tissue development, and prevent protein catabolism has been attempted [13]. Nevertheless, the wide hatching window of chicks of different genotypes resulted in posthatch fasting before being housed on farms. This may have indicated that extra nutrients for muscle contractions and gastrointestinal functioning were needed before the chick emerged from the shell.

Administering exogenous substances has been reported since the 1980s [15], starting with vaccines. As these early developments proceeded, the in ovo technique of applying nutrients through the amniotic sac at the later stages of incubation led to a higher feed conversion ratio (FCR) and improved BW in many cases [16]. Numerous studies were carried out to determine whether in ovo carbohydrates increased protein synthesis in the muscle [17], higher BW, and meat yield. Injecting different carbohydrates was also hypothesized to reduce internal energy consumption, thereby improving hatchability; however, the intervention may be stressful for the late embryo, which would delay hatching time.

Hence, the aim of the study was to examine the effects of a complex carbohydrate solution of disaccharides and glucose administered in ovo on the hatchability and hatching time of differently sized eggs and the development, performance, and carcass characteristics of broilers of different sexes.

## 2. Materials and Methods

### 2.1. Incubation Protocol

The experiment was conducted at the Hungarian University of Agricultural and Life Sciences (MATE) Kaposvár Campus, Department of Farm Animal Nutrition, in accordance with the Declaration of the Hungarian National Scientific Ethical Committee of Animal Experimentation for studies involving animals: protocol license number, SO/31/00956/2020; date of approval, 29 September 2020.

At the beginning of the study, 1200 (Ross × 308) broiler eggs from the same commercial farm (Aviagen Ltd., Mezőörs, Hungary) were examined. They were held in transport boxes for 6 days at 20 °C without rotation or extra humidification due to the short storage time. A PLM B1350 two-staged incubator consisting of nine tray levels was used. Each level was equipped with a mobile measurement system for ventilation, humidity, and temperature among the levels. Hatching protocol was carried out according to the recommendations of the Aviagen Hatching Management Guide (2019); the dry bulb temperature and humidity were set at 37.9 ± 0.1 °C and 65 ± 3%, respectively.

Treatment groups were arranged with 400 eggs separated into three tray levels as a replication. Hatching eggs were categorized as “light” (53–58 g) or “heavy” (>58 g) before incubation. The eggs were candled on day 10 to exclude infertile eggs. The fertility of eggs was calculated after candling by the following formula [18]:

Fertility rate % = number of fertile eggs/total number of eggs set.

On day 17, the eggs were recandled so that the dead embryos could be removed before the in ovo intervention. Completeness of hatching was checked on days 21 and 22, and chicks were collected two times from the incubator on those days. Right after collection, the chicks were sexed according to feather development (fast feathering: pullet; slow feathering: rooster) and given ID-numbered wing tags. The hatching rate was calculated as [18]

Hatch rate % = Number of eggs hatched/total number of eggs set.

### 2.2. Treatment Groups

Three different treatment groups were set, along with a control group (NT). In ovo solutions were prepared on the day of injection and autoclaved at 39 °C for an hour before treatment. Group ioS was injected with 0.5 mL of physiological saline (0.9 g/mL concentration of NaCl), while group ioCH was treated with a carbohydrate complex containing sucrose, maltose, and glucose in a 2:2:1 proportion with 0.5 g/mL concentration dissolved in physiological saline. The number of eggs by egg weight and treatment in the hatching machine is summarized in Table 1.

### 2.3. In Ovo Intervention

The injection procedure was performed in a ScanLaf sterile cabinet (LaboGene Inc., Lillerød, Denmark) to prevent any microbiological contamination, and all eggs had been cleaned with cotton wool dipped in an iodine solution. The in ovo injection was carried out using the Uni and Ferket protocol [16]: a 2 mL syringe with a 21-gauge needle. The eggs were carefully drilled on the blunt side through the air chamber without reaching the shell membrane. Before intervention, the position of the embryos was checked, and afterward, the solutions were transferred to the amniotic fluid. To avoid the entry of pathogens, sterile plastic tape was applied, and the eggs were placed back into the incubator until day 21 of hatching.

### 2.4. Housing Conditions and Feeding Management

The birds were weighed individually at hatch on day 21 or 22 and randomly placed into floor pens (18 birds/pen; 16 pens/treatment). Each pen represented a treatment group; thus, the birds were not mixed within pens. The installation was set up in compliance with EU regulations for temperature, humidity, air movement, harmful gas and dust concentration, hours of light, intensity requirements of livestock, and the recommendations of Aviagen (2019). Air temperature and CO_2_ levels during the hatching are shown in Table 2.

Further live weight measurements were carried out on days 10, 21, and 35. A three-phase feeding program was followed: day 1–10, starter ration (crumbled feed); day 11–21, grower feed; and day 22–35, finisher ration (pelleted feed produced by the Department of Farm Animal Nutrition). Each feed was formulated on a corn–soybean meal basis. Nutritional content––dry matter, crude protein, fat, ash, calcium, and phosphorus—was determined by the University Lab Center of MATE according to the recommendations of the Association of Official Analytical Chemists (AOAC) (2012) [19].

The birds were fed ad libitum from self-feeders during the trial. One feeder was presented per pen. Drinking water was also available ad libitum. The analyzed composition of the feed is presented in Table 3.

Feed intake (FI) was recorded per pen for the time intervals by measuring the offered and remaining feed for each phase. The feed conversion ratio (FCR) was calculated per pen as well. Forty birds randomly selected from each treatment group were slaughtered after 24 h of fasting at the end of the study to examine the effects of the in ovo nutrition-to-carcass characteristics (weight and percentage of thigh and breast muscle compared to liveweight, liver weight, abdominal fat weight and the weight of back and the neck).

### 2.5. Statistical Analysis

Statistical analysis of the experiment was carried out with SAS 9.4 [20]. A Kruskal–Wallis test was carried out on hatching data to determine the effects of in ovo treatments on hatchability and treatment until the day of hatch. Levene’s test was used to examine group homogeneity among treatment groups. A Shapiro–Wilk normality test was carried out on the base data. Then, a randomized block design using a general linear model (GLM) formula was applied to the levels of treatment, sex of the birds, and egg weight to evaluate their effect on growth performance and carcass characteristics. The equation for the performance traits was

*Y* = *X*_1_*β* + *X*_2_*β* + *X*_3_*β* + *X*_4_*β* + *ε*

where *Y* is the dependent variable, *X*_1_ is the explanatory parameter with the fixed effect of treatment, *X*_2_ is the explanatory parameter with fixed effect of sex, *X*_3_ is the explanatory parameter with fixed effect of egg weight, *X*_4_ is the explanatory parameter of the day of hatch (*u*), and ε is the random error. (The interaction of fixed effects is not included in the equation). After applying the model, Tukey’s multiple comparison post hoc tests were applied to determine any differences if the treatment effect was significant (*p* < 0.05). Then, a multiple nonlinear regression was used to predict the contribution of egg weight, day of hatch, hatching weight, and sex to the final weight at 35 days of age in all treatment groups. FI and FCR data were distributed normally; thus, a one-way ANOVA was used with Bonferroni correction to decrease the chance of a Type I error for the LW, average daily gain (ADG), FI, and FCR data.

## 3. Results

### 3.1. Hatchability Results

Of the 1200 eggs, 60 were infertile, and 29 were blood ringed after the first candling (7.46% combined). The numbers for groups NT, ioS, and ioCH were 27, 33, and 29, respectively, before the in ovo intervention on day 10. The remaining 92.54% of the eggs were placed back into the incubator. On day 17, during the second candling, no more eggs were removed. Before the intervention, the number of eggs per treatment was equalized.

The hatching started early in the morning on day 21, and in the afternoon, the hatched birds were collected. Since approximately half of the eggs had still not hatched, the rest of the birds were collected on day 22. The time difference between the two harvestings was approximately 16 h. Overall, 84.83% of the birds were hatched by day 22.

#### 3.1.1. Hatching Rate

**Figure 1:**
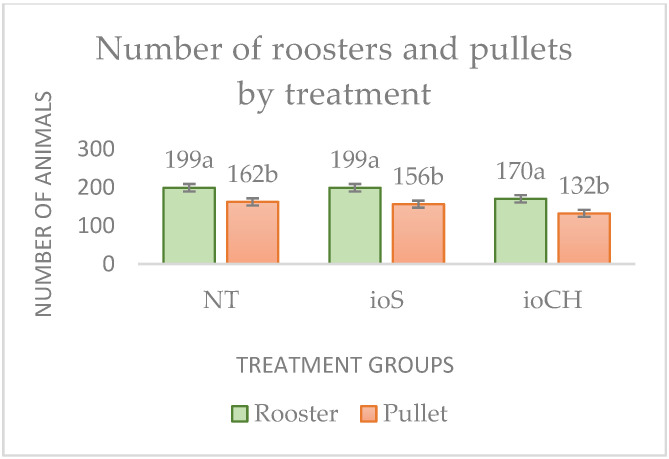
Distribution of hatched chicks by treatment (*p* < 0.001) and sex (*p* < 0.001): NT, control group; ioS, in ovo saline (NaCl); ioCH, in ovo carbohydrate solution. Different letters (a, b) represent significant differences among treatment groups.

**Figure 2:**
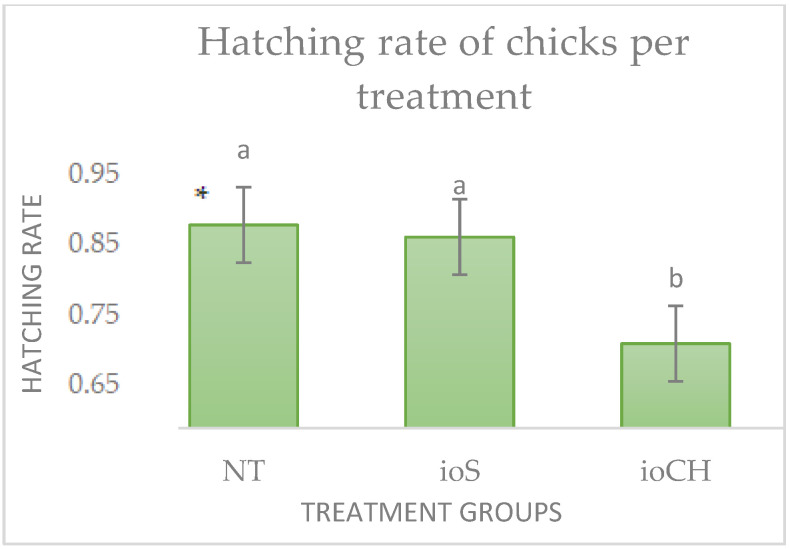
Distribution of hatched chicks by treatment (*p* < 0.001) and sex (*p* < 0.001): NT, control group; ioS, in ovo saline (NaCl); ioCH, in ovo carbohydrate solution. * Error bars represent standard deviations. Different letters (a, b) represent significant differences among treatment groups. Distribution of hatched chicks by treatment (*p* < 0.001) and sex (*p* < 0.001): NT, control group; ioS, in ovo saline (NaCl); ioCH, in ovo carbohydrate solution. * Error bars represent standard deviations. Different letters (a, b) represent significant differences among treatment groups.

The hatching rate was the highest in treatment group NT (90.25%), followed by ioS (88.75%) and ioCH (75.5%). The distribution of hatched chicks by treatment and sex is presented in Figure 1 and Figure 2.

The Kruskal–Wallis test showed the significant stochastic dominance of treatment NT and ioS to hatchability (*p* < 0.001) compared with treatment ioCH. There was no significant difference between groups NT and ioS (*p* = 0.18). The number of roosters was significantly higher (*p* < 0.001) in all treatment groups.

#### 3.1.2. Ratio of Hatched Eggs per Day

**Figure 3:**
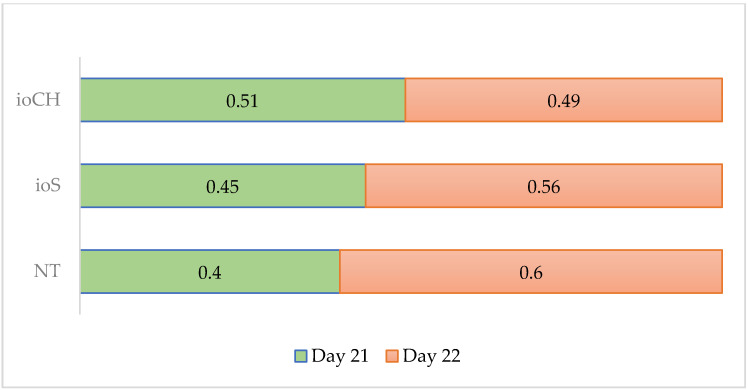
Ratios of hatched eggs per treatment on day 21 and 22: NT, control group; ioS, in ovo saline (NaCl); ioCH, in ovo carbohydrate solution.

The ratio of eggs hatched by day 21 (Figure 3) was favorable in the ioCH group (*p* = 0.04) compared with NT and ioS groups.

### 3.2. Growth Performance

For the performance data, intergroup homogeneity showed a significant difference (*p* = 0.0063). A total of 26 birds were eliminated from the final analysis and the descriptive statistics because their BW was two-standard deviations from the mean and considered “outliers/morbid” from the NT group, while only 10 birds were excluded each from treatment groups ioS and ioCH. The effects of the in ovo treatments are summarized in Table 4 and Table 5.

#### 3.2.1. Liveweight Results

According to the results, the ioCH solution positively influenced the live weight of the birds compared with ioS. There was no significant difference in hatching weight (*p* = 0.3) or live weight at 10 days (*p* = 0.18); however, the possible benefits of the extra energy at the beginning of life resulted in increased body weight later on (*p*-values equal to 0.0082, and 0.0004 on day 21 and 35, respectively). Roosters had significantly higher body weight (*p*-values equal to 0.03, 0.024, and <0.001) except for the hatching weight. Egg weight had a significant effect (*p* < 0.001) on live weight during the trial. Hatching day only affected the weight of the birds from hatch until day 10 (*p* < 0.001). There were significant interactions in the model that revealed that heavy eggs hatching on day 21 showed better starter weight at hatch (egg weight × day of hatch; *p* < 0.001) and maintained this advantage in live weight on days 21 and 35 (*p* = 0.009). Roosters also had favorable hatching weight on day 21. Heavy eggs injected with ioCH had the highest hatching weight. This was also the case for roosters. The greatest live weight results at 10 and 35 days came from chicks hatched at day 21 from heavy eggs with ioCH supplementation (*p* = 0.01).

#### 3.2.2. Average Daily Gain Results

The sex of the birds had a significant effect (*p* < 0.05) on ADG throughout the whole period. The in ovo treatment did not affect the ADG in the starter phase; on the other hand, there was a difference in the ADG between the in ovo saline (*p* = 0.0032) and in ovo carbohydrate treatment groups (*p* = 0.0013) in the grower and finisher phase. Chicks hatched from heavy eggs had better ADG from 22 to 35 (*p* = 0.01). Roosters hatched from heavy eggs and treated with ioCH had favorable ADG between days 1 and 10 and 22 and 35 (*p* = 0.01).

#### 3.2.3. Feed Intake and Feed Conversion Ratio

Differences in FI were only present at the starter phase (*p* = 0.04), in which group ioS differed from group NT. The ioCH solution did not affect the FI. There were no significant differences in FCR over the whole period (*p*-values were equal to 0.54, 0.74, and 0.34 for days 1–10, 11–21, and 22–35, respectively).

**Table 4:** Effects of in ovo treatments, sex, egg weight, and the day of hatch on live weight.

	HW (g)			LW10 (g)			LW21 (g)			LW35 (g)		
**Body Weight per Sex**	**NT**	**ioS**	**ioCH**	**NT**	**ioS**	**ioCH**	**NT**	**ioS**	**ioCH**	**NT**	**ioS**	**ioCH**
Female	43.1	44.2	44.1	185.5	184.2	185.7	676.5	651.4	689.2	1988.7	1909.6	2033.6
Male	43.3	44.1	44.1	183.5	192.8	195.2	709.5	723.9	754.2	2108.3	2088.8	2165.2
**Sex *p*-value**	0.06			**0.04**			0.24			**<0.001**		
**Treatment *p*-value**	0.3			0.18			**0.0082**			**0.0004**		
**Post hoc Tukey’s test results**	**a**	**a**	**a**	**a**	**a**	**a**	**ab**	**a**	**b**	**ab**	**a**	**b**
**Egg weight**												
53–58 g	42	43	42	178.0	179.5	184.4	671.2	667.5	707.3	2003.2	1986.2	2058.7
>58 g	46	46	46	190.8	198.8	197.6	719.7	717.4	744.3	2112.7	2041.4	2160.9
**Egg weight *p*-value**	**<0.001**			**<0.001**			**<0.001**			**<0.001**		
**Day of hatch**												
Day 21	45	45	45	195.2	196.2	192	691.4	700.2	716.2	2038	2005.8	2100
Day22	43	43	42	176.8	183.4	190	697.7	686.2	735.4	2071	2020.1	2117.8
**Day of hatch *p*-value**	**<0.001**			**<0.001**			0.51			0.17		
***p*-values of interactions**												
**Day of hatch** × **Egg weight**	**<0.001**			0.84			**0.009**			**0.009**		
**Day of hatch** × **Sex**	**<0.001**			0.63			0.12			0.46		
**Egg weight** × **Sex**	0.57			0.46			0.55			0.03		
**Day of hatch** × **Trt**	**0.02**			0.056			0.1			0.81		
**Egg weight** × **Trt**	**<0.001**			0.52			0.42			0.43		
**Sex** × **Trt**	0.8			0.14			0.91			0.48		
**Day of hatch** × **Egg weight** × **Sex**	0.15			0.93			0.19			0.12		
**Day of hatch** × **Egg weight** × **Trt**	0.09			**0.01**			0.51			**0.01**		
**Egg weight** × **Sex** × **Trt**	0.88			0.51			0.32			0.22		
**Day of hatch** × **Egg weight** × **Sex** × **Trt**	**0.01**			0.56			0.19			0.32		

HW, hatching weight; LW10, live weight at day 10; LW21, live weight at day 21; LW35, live weight at day 35; NT, control group; ioS, in ovo saline (NaCl); ioCH, in ovo carbohydrate solution. Values in bold represent significant differences, and *p*-values were calculated using the Bonferroni correction. Different letters (a, b) represent significant differences.

**Table 5:** Effects of in ovo treatments on sex, egg weight, and day of hatch to average daily gain.

	ADG 1–10			ADG 11–21			ADG 22–35		
**ADG per Sex**	**NT**	**ioS**	**ioCH**	**NT**	**ioS**	**ioCH**	**NT**	**ioS**	**ioCH**
Female	14.3	14.2	14.3	44.6	42.4	45.7	91.5	88.5	94.7
Male	14.2	15	15.3	47.8	48.3	50.7	98.39	97.2	100.8
**Sex *p*-value**	**0.04**			**<0.001**			**<0.001**		
**Treatment *p*-value**	0.27			**0.0032**			**0.0013**		
**Tukey’s test results**	**a**	**a**	**a**	**a**	**a**	**b**	**ab**	**b**	**a**
**Egg weight**									
53–58 g	13.7	13.9	14.4	44.8	44.3	47.5	93.2	93.24	95.8
>58 g	14.7	15.4	15.3	48	47.1	49.3	97.8	93.9	99.7
**Egg weight *p*-value**	**<0.001**			**<0.001**			**<0.001**		
**Day of hatch**									
Day 21	15.3	15.3	14.9	45.1	45.7	47.6	94.4	92.5	97.6
Day22	13.5	14.1	14.7	47.4	45.7	49.5	96.2	94.4	97.9
**Day of hatch *p*-value**	**<0.001**			**0.02**			**0.09**		
**Interactions**									
**Day of hatch** × **Egg weight**	0.74			0.07			**0.01**		
**Day of hatch × Sex**	0.67			0.4			0.41		
**Egg weight **×** Sex**	0.47			0.08			0.03		
**Day of hatch **×** Trt**	0.07			0.34			0.67		
**Egg weight **×** Trt**	0.51			0.87			0.19		
**Sex **×** Trt**	0.13			0.27			0.46		
**Day of hatch **×** Egg weight **×** Sex**	0.97			0.41			0.12		
**Day of hatch **×** Egg weight **×** Trt**	**0.01**			0.09			**0.01**		
**Egg weight **×** Sex **×** Trt**	0.5			0.24			0.35		
**Day of hatch **×** Egg weight **×** Sex **×** Trt**	0.8			0.36			0.35		

ADG 1–10, average daily gain between days 1 and 10; ADG 11–21, average daily gain between days 11 and 21; ADG 22–35, average daily gain between days 22 and 35; NT, control group; ioS, in ovo saline (NaCl); ioCH, in ovo carbohydrate solution. Values in bold represent significant differences. Different letters (a, b) represent significant differences

Table 6 summarizes the results of feed intake and feed conversion ratio per pen.

#### 3.2.4. Regression Model

The nonlinear multiple regression results (Table 7) showed that treatment (*p* = 0.03), egg weight (*p* = 0.01), and sex (*p* < 0.001) affected the finisher body weight.

### 3.3. Carcass Characteristics

Body weight differences between treatment groups were also expressed in carcass traits (Table 8). The weight of breast (*p* = 0.03) and thigh meat (*p* = 0.004) was significantly higher in group ioCH than in group ioS, while group NT did not differ much from the in ovo-treated birds.

**Figure 4:**
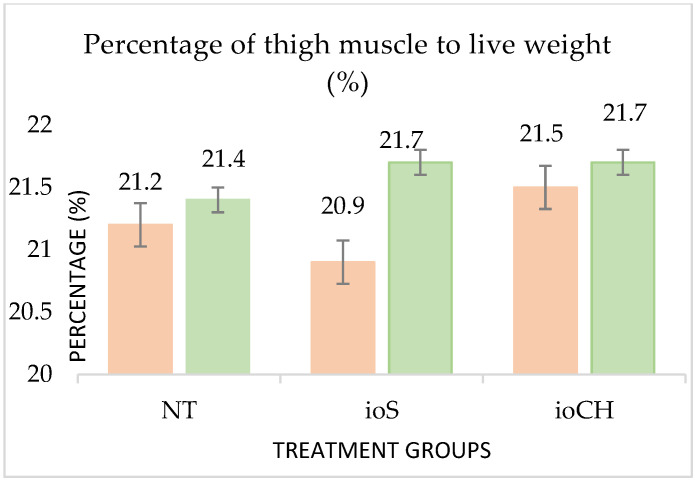
Effects of treatment and sex on thigh (*p* = 0.6 and 0.24) and breast (*p* = 0.01 and 0.4) muscle percentage compared to live weight. Pink columns represent pullets; green columns represent roosters. The error bars represent standard deviations.

**Figure 5:**
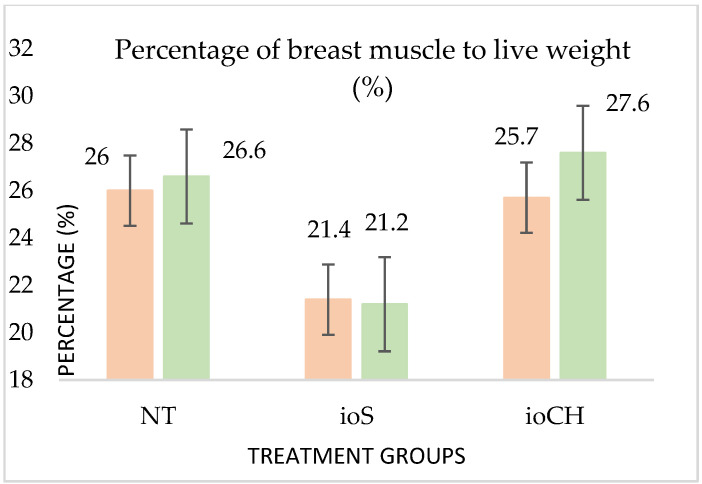
Effects of treatment and sex on thigh (*p* = 0.6 and 0.24) and breast (*p* = 0.01 and 0.4) muscle percentage compared to live weight. Pink columns represent pullets; green columns represent roosters. The error bars represent standard deviations.

The percentage of thigh and breast meat compared to live weight is demonstrated in Figure 4 and Figure 5.

There was no effect on the percentage of thigh muscle regarding sex or treatment (*p*-values are equal to 0.6 and 0.24, respectively); however, the contrast between the sexes and treatments was manifested in the percentage of breast muscle (*p* = 0.01 and 0.04) and also in liver weight (*p* < 0.001), abdominal fat weight (*p* < 0.001), and weight of the back and neck (*p* < 0.001). There were no significant differences between the grill carcasses (calculated by the sum of breast, thigh, back meat, and wings) and the ratios of breast and thigh muscle (breast% and thigh%) compared with the grill carcass among the treatment groups (*p* = 0.6).

## 4. Discussion

In ovo intervention is one of the currently successful methods for overcoming energy deficiency in broiler chickens in the first period of life. Previous studies pointed out that many substances––vitamins, amino acids, minerals, probiotics, and carbohydrates––are able to improve gut health and performance depending on the amount, concentration, genotype of the birds, and the place and time of the injection.

The present study showed that applying in ovo carbohydrates to late-term embryos decreased hatchability by almost 15%. A similar result was found by injecting glucose only [21,22,23] compared with in ovo saline or no injection. Carbohydrate mixtures also tended to reduce the hatching rate [24] and delayed hatching [23]. Previous experiments indicated that to maintain hatchability above 90%, a large quantity of administered fluids should be avoided [23], and the applied concentration should be lower than 25 mg/mL; however, the volume of the solvent is particularly important for minimizing water loss, especially when chicks are transported long distances.

Adding day of hatch to the model, it can be concluded that in ovo injections may accelerate the hatching process (Figure 3) by providing sufficient energy for the embryos and increasing liver glycogen [12]. In some cases, reduced hatchability could also have been a possible effect of injection into the albumen, which may have caused an allergic reaction that stopped the respiration of the embryo. Pedroso et al. [25] reported this outcome after injecting glucose into late-stage broiler embryos, demonstrating that the day of injection combined with a higher amount of a carbohydrate solution also caused hatchability problems. This was also noted by Adriana et al. [26] when eggs were injected with a carbohydrate solution on day 16. Leitao et al. [22] tested glucose at varying levels and concluded that 0.6 mL of glucose solution reduced the hatching rate. Zhai et al. [27] achieved the same result with 0.4 mL of carbohydrate solution. The low rates of water loss itself might have caused hatchability problems by delayed hatching or even failing to hatch because an egg must lose approximately 12–15% of its weight to the point of pipping [28].

From examining the performance results, hatching weight was not influenced by the in ovo intervention; however, carbohydrate excess was expected to increase energy storage of the embryo and enhance hatching weight, as reported by others [29,30,31]. Previous attempts successfully described that maltose appeared to bring better results for hatching weight [32], but this did not occur when maltose was added to a carbohydrate mixture. Looking at the interactions of Table 2, ioCH treatment with heavy eggs caused better nutrient use of the yolk, resulting in better live weight records later on. Similar findings were recorded by Enting et al. (2007) [33]. The combination of maltose, sucrose, and dextrin effectively amplified the intestinal villi surface, leading to increased BW at 10 days, along with liver glycogen and pectoral muscle size [34]. The ability to digest disaccharides was also enhanced by in ovo carbohydrates, resulting in a higher finisher weight [35]. Favorable developmental status of the gastrointestinal tract from the in ovo carbohydrate treatment indicated a higher BW of chicks in the fattening period [28]. In our study, in ovo injection of a carbohydrate mixture resulted in 124 g more BW for females and 77 g for males on day 35 compared with the ioS treatment (Table 2). Similar increased BW results were observed in ducks (*Anas platyrhynchos domestica*) from in ovo sucrose and maltose injection and in turkeys (*Meleagris gallopavo domesticus*) from in ovo glucose [36].

The applied carbohydrate mixture had no effect on the ADG at the starter phase, as reported by Bhanja et al. [37]; however, the authors also experienced higher glucose and protein levels in the plasma with lower uric acid by day 10, concluding that glucose contributed to better intestinal development. Increased ADG at the grower and finisher phase of the ioCH group compared with the ioS treatment revealed that depletion of glycogen stores during hatching negatively affected growth despite the water excess. The positive effects of in ovo glucose on ADG were previously noted by several authors [31,32,38]. Adding disaccharides to broiler embryos also enhanced digestion and growth [38]. Higher FI in group ioS in the first period of life demonstrated the consequences of energy deficit and stress reactions from the needle puncture, which were compensated for during fattening. A comparative study [2] examining 17 papers also concluded that in ovo carbohydrate solutions resulted in better FI, in one case [31] by injecting 0.5 mL of 15 and 20% glucose into the albumen on day 7.

Looking at the improved slaughter performance of group ioCH (5.4% breast muscle percentage, representing 72 g in muscle weight) compared with group ioS, the present study refers to the early stimulation of the gastrointestinal tract that was also reported by Kucharska–Gaca et al. [39] It led to greater muscle satellite cell proliferation due to protein accumulation. Similar results were observed in pectoral muscle weight and carcass yield by adding in ovo β-hydroxy-β-methylbutyrate, which supported the energy status of the birds prior to hatching [35]. Confirming the positive effect of carbohydrates, it was stated that applying a glucose and magnesium mixture during incubation successfully improved carcass yield and posthatch performance [31]. These findings confirm that feeding in ovo carbohydrates may be useful for improving broiler performance; however, a large number of factors may affect the final results. Future directions could focus on the method such as the injection site on the egg, solution amount, and osmolality.

## 5. Conclusions

The current results confirm that carbohydrate mixtures for late broiler embryos can decrease hatchability, which is, according to the literature, related to the osmolarity of the solution. In the case of in ovo intervention with carbohydrates, the emergence of the birds from the egg shifted to an earlier date. However, it can be concluded that in ovo carbohydrate supplementation can be beneficial for male broilers if they have access to feed approximately 36 h after hatching. The intervention improved body weight during fattening and improved the number of valuable meat parts at slaughter. These findings indicated that further studies are needed to determine the ideal concentration of in ovo carbohydrate supplements for optimal hatchability and raising farm profit.

## Figures and Tables

**Table 1:** Treatment groups in the hatching machine.

Tray Levels	Egg Weight	Treatment Groups	Number of Eggs
1	Light	ioCH	66
	Heavy		67
2	Light	ioS	67
	Heavy		66
3	Light	NT	67
	Heavy		67
4	Light	ioCH	67
	Heavy		67
5	Light	ioS	66
	Heavy		67
6	Light	NT	66
	Heavy		67
7	Light	ioCH	67
	Heavy		66
8	Light	ioS	67
	Heavy		67
9	Light	NT	66
	Heavy		66

**Table 2:** Temperature and CO_2_ levels during incubation.

Hatching Day	°C	% CO_2_
**1**	37.9	0.60
**2**	37.9	0.60
**3**	37.9	0.60
**4**	37.9	0.60
**5**	37.9	0.60
**6**	37.9	0.60
**7**	37.8	0.60
**8**	37.8	0.60
**9**	37.6	0.60
**10** **Candling**	37.6	0.60
**11**	37.5	0.35
**12**	37.5	0.35
**13**	37.4	0.35
**14**	37.3	0.35
**15**	37.3	0.35
**16**	37.2	0.35
**17 Candling,** **in ovo intervention, placing into the incubator**	37.1	0.35
**18**	37.0/36.7	0.35/0.60
**19**	36.7	0.60
**20**	36.5	0.60
**21**	36.2	0.60
**22**	36.2/35.8	0.35

**Table 3:** Composition and calculated nutrient content of the basal diets (g/kg).

Ingredients	Starter (1–10)	Grower (11–21)	Finisher (22–35)
Corn (grain)	551	577	601
Corn gluten (60%)	32	32	32
Sunflower meal	53.5	53.5	75
Soybean meal (CP 44.2%)	262	230	175
Fat, vegetable	44.7	55	67.00
MCP	18.7	17.5	15
Limestone	15	13.5	12.2
NaCl	2.7	2.7	2.7
L-Lysin HCl	5.2	4.6	4.3
DL-Methionin	4.5	3.9	3.2
L-Treonin	2.6	2.3	1.8
Premix ^1^	5.00	5.00	5.00
**Total**	**1000.00**	**1000.00**	**1000.00**
**Nutrient content (g/kg)**			
AMEn (MJ/kg)	12.5	12.9	13.4
DM %	90	91.3	91.1
Crude protein	204.2	190.7	174.9
Crude fat	71.87	82.3	94.4
Crude fiber	41.5	41.1	44.8
Lysine	13.5	12,1	10,8
M + C	10.8	9.9	9.0
Threonin	9.7	8,8	7,8
Tryptophan	2.4	2.3	1.7
Ca	9.6	8.7	7.8
P_available_	4.7	4.5	3.9
Na	1.7	1.7	1.7

^1^ Premix feed contents per kilogram: Zn, 22,032 mg; Cu, 3200 mg; Fe, 16,020 mg; Mn, 21,948 mg; I, 300 mg; Se, 70 mg; Co, 20 mg; Vit. A, 3,240,000 IU; Vit. D3, 810,000 IU; Vit. E, 20,800 mg; Vit. K3, 810 mg; Vit. B1, 810 mg; Vit. B2, 1890 mg; Vit. B3, 10,800 mg; Vit. B5, 3240 mg; Vit. B6, 1350 mg; Vit B12, 6.8 mg; folic acid, 270 mg; biotin, 32 mg.

**Table 6:** Feed intake (FI) and feed conversion ratio (FCR) of broiler chicken.

Trt	FI 1–10	FI 11–21	FI 22–35	FI Total	FCR 1–10	FCR 11–21	FCR 22–35	FCR Total
**NT**	19.2 a	67.9	138.4	81.8	1.56	1.68	1.70	1.68
**ioS**	20.4 b	66.0	139.7	82.2	1.6	1.66	1.73	1.70
**ioCH**	19.7 ab	66.9	141.7	83.2	1.57	1.63	1.71	1.68
***p*-value**	**0.04**	0.54	0.65	0.74	0.54	0.74	0.34	0.54
**RMSE**	1.42	5.03	8.86	4.55	0.12	0.15	0.07	0.06

Trt, treatment groups; NT, control group; ioS, in ovo saline (NaCl); ioCH, in ovo carbohydrate solution; FI, feed intake of the birds in between the feeding phases measured by pen; FCR, feed conversion ratio of the birds in between the feeding phases measured by pen. Values in bold represent significant differences. Different letters (a, b) represent significant differences.

**Table 7:** Parameters of the multiple regression procedure to evaluate the main effects on the final body weight.

Variable	Parameter Estimate	SE	t-Value	*p*-Value
**Intercept**	1791.78	219.00	8.18	<0.0001
**Treatment**	26.03	12.23	2.13	**0.03**
**Egg weight**	64.83	26.08	2.49	**0.01**
**Day of hatch**	39.87	23.08	1.73	0.08
**Sex**	−142.22	19.85	−7.16	**<0.0001**
**Hatching weight**	6.50	5.01	1.3	0.19
**RMSE**	276.24			
**R^2^**	0.91			
**Adjusted R^2^**	0.86			

Values in bold represent significant differences.

**Table 8:** Thigh and breast muscle weight.

		Thigh (g)			Breast (g)	
**Weight of Breast and Thigh per Sex**	**NT**	**ioS**	**ioCH**	**NT**	**ioS**	**ioCH**
Male	400.8	408.7	410.5	492.4	452.9	522.8
Female	405.1	388.0	422.0	505.0	465.8	500.3
**Trt *p*-value**	**0.004**			**0.03**		
**Tukey’s test results**	**ab**	**a**	**b**	**ab**	**a**	**b**
**Sex *p*-value**	0.88			0.13		

Trt, treatment groups; NT, control group; ioS, in ovo saline (NaCl); ioCH, in ovo carbohydrate solution. Different letters (a, b) represent significant differences.

## Data Availability

The data presented in this study are available on request from the corresponding author. The study did not include humans.

## References

[1] Zuidhof M.J., Schneider B.L., Carney V.L., Korver D.L., Robinson F.E. (2014). Growth, Efficiency, and Yield of Commercial Broilers from 1957, 1978, and 2005. Poult. Sci..

[2] Retes P.L., Clemente A.H.S., Neves N.G., Espósito M., Makiyama L., Alvarenga R.R. (2017). In ovo feeding of carbohydrates for broilers—A systematic review. J. Anim. Phys. Anim. Nut..

[3] Noble R.C., Cocchi M. (1990). Lipid metabolism and the neonatal chicken. Prog. Lipid Res..

[4] Boersma S.I., Robinson F.E., Renema R.A., Fasenko G.M. (2003). Administering oasis hatching supplement prior to chick placement increases initial growth with no effect on body weight uniformity of female broiler breeders after three weeks of age. J. Appl. Poult. Res..

[5] De Oliveira J.E., Uni Z., Ferket P.R. (2008). Important metabolic pathways in poultry embryos prior to hatch. World’s Poult. Sci. J..

[6] Clark D., Sokoloff L. (1999). Basic Neurochemistry: Molecular, Cellular and Medical Aspects.

[7] Christensen V.L., Wineland M.J., Fasenko G.M., Donaldson W.E. (2001). Egg storage effects on plasma glucose and supply and demand tissue glycogen concentrations of broiler embryos. Poult. Sci..

[8] Pearce J. (1971). Carbohydrate metabolism in the domestic fowl. Proc. Nutr. Soc..

[9] Klasing K.C. (1998). Comperative Avian Nutrition.

[10] Pearce J., Brown W.O., Bell D.J., Freeman B.M. (1971). Carbohydrate Metabolism in Physiology and Biochemistry of the Domestic Fowl.

[11] Burley R.W., Vadehra D.V. (1989). The Avian Egg. Chemistry and Biology.

[12] Berg J.M., Tymoczko J.L., Stryer L. (2002). Biochemistry.

[13] Moran E.T. (2007). Nutrition of the developing embryo and hatchling. Poult. Sci..

[14] Sklan D. (2001). Development of the digestive tract of poultry. Worlds Poult. Sci. J..

[15] Sharma J., Burmester B. (1982). Resistance of Marek's disease at hatching in chickens vaccinated as embryos with the turkey herpesvirus. Avian Dis..

[16] Uni Z., Ferket P.R. (2003). Enhancement of Development of Oviparous Species by in Ovo Feeding. U.S. Regular Patent.

[17] EI-Husseiny O.M., EI-Wafa S.A., EI-Komy H.M.A. (2008). Influence of fasting or early feeding on broiler performance. Int. J. Poult. Sci..

[18] Alasahan S., Copur A.G. (2016). Hatching characteristics and growth performance of eggs with different shapes. Braz. J. Poult. Sci..

[19] (2013). Official Methods of Analysis of AOAC International.

[20] SAS Institute Inc. (2013). SAS/ACCESS^®^ 9.4 Interface to ADABAS: Reference.

[21] Pedroso A.A., Chave L.S., Lopes K.L.A.M., Leandro N.S.M., Café M.B., Stringhini J.H. (2006). Inoculação de nutrientes em ovos de matrizes pesadas. Rev. Bras. Zootec..

[22] Leitão R.A., Leandro N.S.M., Café M.B., Stringhini J.H., Pedroso A.A., Chaves L.d.S. (2008). Inoculação de glicose em ovos embrionados de frango de corte: Parâmetros de incubação e desempenho inicial. Cienc. Anim. Bras..

[23] Zhai W., Rowe D.E., Peebles E.D. (2011). Effects of commercial in ovo injection of carbohydrates on broiler embryogenesis. Poult. Sci..

[24] Campos A.M.A., Rostagno H.S., Gomes P.C., Silva E.A., Albino L.F.T., Nogueira E.T. (2011). Efeito da inoculação de soluções nutritivas in ovo sobre a eclodibilidade e o desempenho de frangos de corte. Rev. Bras. Zootec..

[25] Chotinsky D., Toncheva E., Profirov Y. (2001). Development of dissacharidases activity in the small intestine of broiler chickens. Br. Poult. Sci..

[26] Pedroso A.A., Chaves L.S., de Almeida Martinez Lopes K.L., Leandro N.S.M., Café M.B., Stringhini J.H. (2006). Nutrient inoculation in eggs from heavy breeders. Rev. Bras. Zootec..

[27] Zhai W., Gerard P.D., Pulikanti R., Peebles E. (2011). Effects of in ovo injection of carbohydrates on embryonic metabolism, hatchability, and subsequent somatic characteristics of broiler hatchlings. Poult. Sci..

[28] Romanoff A.L. (1930). Biochemistry and biophysics of the developing hen’s egg. I. Influence of humidity. Mem. Cornell Univ. Agric. Exp. Stn..

[29] Shafey T.M., Alodan M.A., Al-Ruqaie I.M., Abouheif M.A. (2012). In ovo feeding of carbohydrates and incubated at a high incubation temperature on hatchability and glycogen status of chicks. S. Afr. J. Anim. Sci..

[30] Salmanzadeh M. (2012). The effects of in-ovo injection of glucose on hatchability, hatching weight and subsequent performance of newly-hatched chicks. Rev. Bras. Ciência Avícola.

[31] Salmanzadeh M., Ebrahimnejad Y., Shahryar H.A., Gorbani A., Oskuei H.R. (2011). The effects of in ovo glucose administration on hatching results and subsequent blood glucose concentration in newly-hatched chicks. J. Appl. Biol. Sci..

[32] Enting H., Kruip T.A.M., Verstegen M.W.A., van Der Aar P.J. (2007). The effect of low-density diets on broiler breeder performance during the laying period and on embryonic development of their offspring. Poult. Sci..

[33] Kornasio R., Halevy O., Kedar O., Uni Z. (2011). Effect of in ovo feeding and its interaction with timing of first feed on glycogen reserves, muscle growth, and body weight. Poult. Sci..

[34] Uni Z., Ferket P.R., Tako E., Kedar O. (2005). In ovo feeding improves energy status of late-term chicken embryos. Poult. Sci..

[35] Chen W., Wang R., Wan H.F., Xiong X.L., Peng P., Peng J. (2009). Influence of in ovo injection of glutamine and carbohydrates on digestive organs and pectoralis muscle mass in the duck. Brit. Poult. Sci..

[36] Amitav B., Majumdar S., Bhanja S.K., Mandal A.B., Dash B.B., Agarwal S.K. Effect of in ovo injection of glucoseon growth, immunocompetence and development of diges-tive organs in turkey poults. Proceedings of the 16th European Symposium on Poultry Nutrition.

[37] Bhanja S.K., Mandal A.B., Agarwal S.K., Majumdar S. (2008). Effect of in ovo glucose injection on the post-hatch growth, digestive organ development and blood biochemical profilesin broiler chickens. Indian J. Anim. Sci..

[38] Tako E., Ferket P.R., Uni Z. (2004). Effects of in ovo feeding of carbohydrates and beta-hydroxy-beta-methylbutyrate on the development of chicken intestine. Poult. Sci..

[39] Kucharska-Gaca J., Kowalska E., Dębowska M. (2017). In ovo Feeding-Technology of the Future—A Review. Ann. Anim. Sci..

